# Proteomic analysis of watery saliva secreted by white-backed planthopper, *Sogatella furcifera*

**DOI:** 10.1371/journal.pone.0193831

**Published:** 2018-05-04

**Authors:** Yu-Tong Miao, Yao Deng, Hao-Kang Jia, Yu-Di Liu, Mao-Lin Hou

**Affiliations:** 1 State Key Laboratory for Biology of Plant Disease and Insect Pests, Institute of Plant Protection, Chinese Academy of Agricultural Sciences, Beijing, China; 2 Scientific Observing and Experimental Station of Crop Pests in Guilin, Ministry of Agriculture, Guilin, China; Zhejiang University, CHINA

## Abstract

The white-backed planthopper, *Sogatella furcifera*, is a phloem sap feeder that secretes watery and gelling saliva during feeding. In this study, we identified the major proteins in watery saliva of *S*. *furcifera* by shotgun LC-MS/MS analysis combined with transcriptomic analysis. A total of 161 proteins were identified, which were divided into 8 function categories, including enzymes, transporter, calcium ion binding protein, salivary sheath protein, cytoskeleton protein, DNA-, RNA-, and protein-binding or regulating proteins, other non-enzyme proteins and unknown proteins. Gene expression pattern of 11 secretory proteins were analyzed by real time quantitative-PCR. We detected the mucin-like protein, which had a unique expression level in salivary gland, most likely to be a candidate effector involved in regulation of plant defense. This study identified the watery saliva component of *S*. *furcifera* and it provided a list of proteins which may play a role in interaction between *S*. *furcifera* and rice.

## Introduction

Saliva is an important biochemical interface determining the compatibility between sap-sucking insects and their host plants [1, 2]. The saliva includes bioactive compounds that have a range of functions from degrading plant cell and digesting nutrients to eliciting or inhibiting plant defense [3]. Hemipterans secrete two types of saliva: gelling saliva and watery saliva. Gelling saliva is used to form salivary sheath in order to facilitate stylet penetration, while watery saliva involves in the regulation of plant defense [4].

White-backed planthopper, *Sogatella furcifera*, a critical phloem sap feeder, causes considerable damage by sucking plant sap and transmitting plant viral diseases [5]. It also secretes gelling and watery saliva during feeding process like other hemipterans. Previous studies have detected the saliva composition in many species of aphids [1, 6–9], *Nephotettix cincticeps* [10], *Lygus Hesperus* [11] and *Nilaparvata lugens* [4, 12]. Huang et al. [13] compared the secreted saliva composition of three planthopper species and revealed the ubiquitous and specific saliva compounds in different insects. Li et al. [14] sequenced and assembled the transcriptome of *S*. *furcifera* salivary gland, laying the foundation of research on saliva of *S*. *furcifera*.

In this study, shotgun liquid chromatography-tandem mass spectrometry (LC-MS/MS) was carried out to identify the watery saliva component of *S*. *furcifera*, identification of salivary protein was combined with transcriptome of *S*. *furcifera* and *N*. *lugens* salivary gland. We quantified the relative expression of predicted secretory proteins and detected many salivary proteins that may modulate plant defense. This study exhibits an overview of *S*. *furcifera* salivary proteins and makes it possible to understand the mechanism of interaction between white-backed planthopper and rice. Moreover, we make an attempt to exploit the candidate effectors in *S*. *furcifera*, which may provide a new target for pest management.

## Materials and methods

### Insects

The *S*. *furcifera* populations were originally collected from Xing'an County, Guilin, Guangxi Zhuang Autonomous Region, China, in 2013, which is the Scientific Observing and Experimental Station of Crop Pests of Guilin/Guilin Branch, Institute of Plant Protection, Chinese Academy of Agricultural Sciences. The field studied did not involve endangered or protected species, and no specific permissions were required for these activities in this station. The insects were reared on susceptible rice TN1. The colony was maintained in insect cages in a thermostat at 27 ± 1°C and 80 ± 10% RH, under a 16:8 L: D photoperiod.

### Collection and concentration of watery saliva

Each saliva collection container was prepared by stretching two layers of Parafilm over one side of a sterile glass tube (4 cm × 8 cm) with 200 μL 15% sucrose diet. 50 *S*. *furcifera* adults were held in each tube and we collected 8 000 adults in total. To attract the insects and keep humid, another side of the tube was wrapped up with a piece of wet black cloth. The collection tubes were placed in a thermostat at 27 ± 1°C and 80 ± 10% RH, under a 16:8 L: D photoperiod. After 24 h of stylet-probing and salivary discharging by the insects, collection tubes were removed from the thermostat. Diet was collected under sterile conditions by making a small incision in the Parafilm with an injector needle and pouring the stylet-probed diet into sterile centrifuge tubes with pipette. Saliva collection was conserved under -80°C.

The collection of saliva was centrifuged at 7 000 g at 4°C for 30 min to remove the impurity and deposit. The supernatant was ultrafiltered with 3 kDa molecular weight cut offs (Amicon-4 Ultra; Millipore, MA, USA), and then concentrated by adding fivefold volume of trichloroacetic acid/acetone (1/9) solution. After incubating at -20°C for 4 h and centrifuging at 6 000 g at 4°C for 40 min, the supernatant was removed and the precipitation was washed 3 times with chilled acetone. Sample was dissolved in SDT buffer (4% sodium dodecylsulfate (SDS, Bio-Rad, Hercules, CA, USA), 100 mM Tris-HCl, 1 mM dithiothreitol (DTT, Bio-Rad), pH 7.6) and incubated in hot water for 15 min and centrifuged at 14 000 g for 40 min.

### Protein digestion

Filter-aided sample preparation (FASP) method [15] was used for protein digestion. 30 μL protein sample was added to 100 mM DTT and incubated in hot water for 5 min. After adding 200 μL UA buffer (8 M urea (Bio-Rad), 150 mM Tris-HCl, pH 8.0), the mixture was transferred to 10 kDa ultrafiltration centrifuge tube (Sartorius, Gottingen, Germany) and centrifuged twice at 14 000 g for 15 min. 100 μL IAA buffer (100 mM IAA in UA) was added to the tube and incubated in dark for 30 min at room temperature after vortexing for 1 min at 600 rpm. Then centrifuge at 14 000 g for 15 min. Sample was added 100 μL UA buffer and centrifuged at 14 000 g for 15 min. Perform this procedure twice. Then sample was digested with 1 μg trypsin (Promega, Madison, USA) in 40 μL 100 mM NH_4_HCO_3_ (Sigma, St. Louis, MO, USA) buffer. After vortexing for 1 min at 600 rpm, the mixture was reacted for 16–18 h at 37°C. Sample was centrifuged at 14 000 g for 15 min and transferred to a new collection tube. 40 μL 25 mM NH_4_HCO_3_ was added to the tube and then centrifuged at 14 000 g for 15 min to collect filtrate. C18 Cartridge (Sigma) was used to desalinate the peptides. 15 μL 0.1% formic acid (FA, Sigma) was added after lyophilizing the peptides. Peptides were qualified at OD_280_.

### LC/MS-MS analysis

LC/MS-MS was performed on an Easy nLC (Thermo Fisher Scientific, MA, USA) coupled with Q Exactive mass spectrometer (Thermo Fisher Scientific). 6 μL digested peptides were used for LC/MS-MS analysis. The mobile phases A was 0.1% FA/H_2_O and mobile phases B was 0.1% FA/H_2_O and 84% acetonitrile (ACN, Merck, Darmstadt, Germany)/H_2_O. The peptides were loaded in C18-reversed phase column (Thermo Scientific Acclaim PepMap100, 100 μm × 2 cm, nanoViper C18) and separated in analytical column (Thermo scientific EASY column, 10 cm, ID 75 μm, 3 μm, C18-A2) at a flow rate of 300 nL/min. The liquid phase gradient was as follows: 0% B to 55% B at 0–110 min, 55% B to 100% B at 110–115 min and 100% B at 115–120 min.

Peptide separations were analyzed using a Q Exactive mass spectrometer by dynamically choosing the 20 most abundant ions from one full mass scan (300–1800 m/z) for high-energy collisional dissociation (HCD) fragmentation. Normalized collision energy was 27 eV and dynamic exclusion was 60 s. Under fill ratio was defined as 0.1%. First level of mass spectrum resolution was 70 000 at m/z 200 and second level was 17 500 at m/z 200. Automatic gain control (AGC) target was 3e6.

### Protein identification

MS/MS spectra were searched using Maxquant software (Max Planck Institute of Biochemistry in Martinsried, Germany, version 1.3.0.5) against three databases: (1) the public Uniprot database with parameters set for Auchenorrhyncha (http://www.uniprot.org, 16 168 coding protein sequences); (2) transcriptomic database of *S*. *furcifera* salivary gland (http://www.ncbi.nlm.nih.gov/sra, accession number SRR3211109); and (3) transcriptomic database of *N*. *lugens* salivary gland (http://www.ncbi.nlm.nih.gov/sra, accession number SRR5149721). For protein identification, search parameters were as follows: (1) enzyme, trypsin; (2) max missed cleavages, 2; (3) MS/MS tolerance, 20 ppm; (4) fixed modifications, carbamidomethyl (C); (5) variable modifications, oxidation (M), acetyl (protein N-term); (6) database pattern: reverse; (7) protein and peptide false discovery rate (FDR), ≤0.01.

### Bioinformatic analysis

Gene Ontology (GO) annotations of identified proteins were assigned according to information available in the Swiss-Prot/TrEMBL (http://www.uniprot.org/) and Gene Ontology database (http://geneontology.org/). MS-identified proteins were categorized by molecular function, biological process and cellular component. Kyoto Encyclopedia of Genes and Genomes (KEGG) pathway of identified proteins was performed by BlastKOALA in KEGG database (http://www.kegg.jp/). Signal peptide was determined by SignalP 4.1 Server (http://www.cbs.dtu.dk/services/SignalP/). Subcelluar localization was predicted by TargetP 1.1 Server (http://www.cbs.dtu.dk/services/TargetP/). THMHH Server v. 2.0 (http://www.cbs.dtu.dk/services/ TMHMM/) was used to predict transmembrane helices in proteins. Protein domains were determined by Pfam version 31.0 (http://pfam.xfam.org/).

### Prediction of secretory protein and amplification of candidate sequence

Probable secretory proteins were predicted by signal peptide and transmembrane domain. In eukaryotes, there are two cases for secretory proteins: (1) presence of a signal peptide and absence of transmembrane domain; (2) presence of a signal peptide and a transmembrane domain, and meanwhile, the transmembrane domain was in range of the signal peptide [9]. Proteins qualified the conditions were searched in the transcriptomic database of *S*. *furcifera* salivary gland and verified using blastx to search for similar sequences in NCBI non-redundant protein database with default parameters. The open reading frame (ORF) of each candidate gene was determined using the ExPASY Translate Tool (http://web.expasy.org/translate/). Gene-specific primers (S1 Table) designed by Primer Premier 5.0 were used to clone the complete ORF or partial sequences of each salivary protein gene. The total RNA from *S*. *furcifera* adult was extracted by using TRIzol Reagent (Invitrogen, MA, USA) according to manufacturer’s instructions. Template cDNA was synthesized using the Fast Quant RT kit (TIANGEN, Beijing, China). The PCR products were cloned into the pEASY-T1 vector (TransGen Biotech, Beijing, China) and the insert was sequenced with standard M13 primers.

### Real-time quantitative PCR (qPCR) for gene expression analysis

To investigate the tissue-, developmental stage- and sex-specific expression patterns of *S*. *furcifera* secretory protein genes, real-time qRCR was conducted using an ABI 7500 Real-Time PCR System (Applied Biosystems, Carlsbad, CA, USA). The tissues including salivary gland, head (without salivary gland), gut, testis, ovary and remaining body were dissected from *S*. *furcifera* adults under an anatomical lens (Leica Microsystems GmbH, Wetzlar, Germany) with microforceps (Shanghai Medical Instruments Ltd., Corp.) in chilled 1×Phosphate Buffered Saline (1×PBS) solution (pH 7.2, Life Technologies Corporation, NY, USA). Different developmental stages of *S*. *furcifera* including egg, 1^st^-2^nd^ instar nymphs, 3^rd^-4^th^ instar nymphs, 5^th^ instar nymphs, newly emerged female and male adults, and female and male adults after molting for 5 days were collected. For tissues, total RNA was isolated using the PureLink RNA Mini Kit (Life Technologies, Carlsbad, CA, USA). Total RNA of the whole insect was extracted by using TRIzol Reagent (Invitrogen).

Gene-specific primers (S2 Table) were designed using primer3 web (version 4.0.0) (http://primer3.ut.ee/) and Beacon Designer 7.90, and the cDNA was prepared according to the instruction. The *S*. *furcifera* housekeeping gene Ribosomal protein L9 (GeneBank accession number KP735523) and Ribosomal protein L10 (GeneBank accession number KP735524) were used as internal control [16]. The specificity and efficiency of each primer was validated by analyzing standard curves with a fivefold cDNA dilution series.

Each qPCR reaction was conducted in a 20 μl mixture containing 10 μl of Bester^®^ SybrGreen qPCR mastermix (DBI^®^ Bioscience, Germany), 0.4 μl of each primer (10 μM), 0.04 μl of 50 × ROX Reference Dye, 4 μl of sample cDNA and 5.16 μl of sterilized H_2_O. The first-strand cDNA and a no-reverse-transcription control were used as templates for three biological replicates under the following reaction program: an initial denaturation step at 95°C for 5 min, followed by 40 cycles of 95°C for 10 s and 60°C for 31 s, melt curves stages at 95°C for 15 s, 60°C for 1 min, and 95°C for 15 s. Relative quantification of salivary protein genes in different tissues and developmental stages was performed with the 2^-ΔΔCt^ method [17]. Data analysis was performed using the SPSS Statistics 20.0 software (IBM SPSS Statistics Inc., Chicago, IL, USA). A one-way nested analysis of variance (ANOVA) and Duncan’s multiple range test (*p* < 0.05) were used to calculate the relative expression of each target gene. The values were presented as the mean ± SE when applicable.

## Results

### Identification of proteins of watery saliva in *S*. *furcifera*

A total of 161 proteins were identified from *S*. *furcifera* secreted watery saliva by shotgun LC-MS/MS analysis (Table 1). According to their functions, the identified proteins were divided into 8 categories: (1) enzymes including oxidoreductases, hydrolases, peptidases, proteases, transferases, lyases, isomerases, ligases and ATP synthases; (2) transporter including ABC transporter, endoplasmic reticulum (ER) -Golgi transporter, ion transporter, Golgi transporter, lipid transporter, lysosome transporter, protein transporter and vacuolar transporter; (3) calcium ion binding protein; (4) putative sheath protein; (5) cytoskeleton protein; (6) DNA-, RNA-, and protein-binding or regulating proteins; (7) other non-enzyme proteins such as ubiquitin, antigen, chaperone protein, ribosomal protein, heat shock protein and signal transduction protein; (8) unknown protein.

**Table 1:** Summarizes the proteins of watery saliva of *S*. *furcifera* identified by LC-MS/MS.

Protein identification	No. of unique peptides^a^	Protein domain^b^	THMHH^c^	TargetP^d^	SignalP^e^	Function group
Cytochrome c oxidase subunit 1	22	Cytochrome C and Quinol oxidase polypeptide I	12	S	Yes	Oxidoreductases
NADH-ubiquinone oxidoreductase chain 6	2	plastoquinone oxidoreductase chain 6	4	S	Yes	Oxidoreductases
NADH-ubiquinone oxidoreductase chain 3	3	NADH-ubiquinone/plastoquinone oxidoreductase, chain 3	3	S	Yes	Oxidoreductases
NADH-ubiquinone oxidoreductase chain 2	1	Proton-conducting membrane transporter	5	S	Yes	Oxidoreductases
Cytochrome P450 CYP419A1	1	Cytochrome P450	0	/	No	Oxidoreductases
Cytochrome b	3	Cytochromeb (C-terminal)/b6/petD Cytochrome b/b6/petB; Cytochrome b(N-terminal)/b6/petB	4	S	No	Oxidoreductases
Cytochrome c oxidase subunit 2	1	Cytochrome C oxidase subunit II, periplasmic domain; Cytochrome C oxidase subunit II, transmembrane domain	2	S	No	Oxidoreductases
NADH dehydrogenase subunit 6	1	/	4	S	Yes	Oxidoreductases
Peptidylglycine α-hydroxylating monooxygenase	1	Copper type II ascorbatedependent monooxygenase, C-terminal domain; Copper type II ascorbate-dependent monooxygenase, N-terminal domain	1	S	Yes	Oxidoreductases
Inosine-5'-monophosphate dehydrogenase	1	IMP dehydrogenase / GMP reductase domain; CBS domain	0	/	No	Oxidoreductases
Maestro heat-like repeat-containing protein family member 1	1	/	0	S	No	Oxidoreductases
Aldo-keto reductase	1	Aldo/keto reductase family	0	/	No	Oxidoreductases
Uncharacterized protein LOC100165697	1	/	0	/	No	Oxidoreductases
Isolate 9 chitinase	1	Glycosyl hydrolases family 18	0	M	No	Hydrolases
Carboxylesterase	2	Carboxylesterase family	0	S	Yes	Hydrolases
Non-lysosomal glucosylceramidase	1	Glycosyl-hydrolase family 116, catalytic region; beta-glucosidase 2, glycosyl-hydrolase family 116 N-term	1	/	No	Hydrolases
Dicer 2	1	PAZ domain; Ribonuclease III domain	0	/	No	Hydrolases
Ankyrin repeat domain-containing protein	1	Ankyrin repeats (3 copies); Ankyrin repeats (many copies)	0	/	No	Hydrolases
Uncharacterized protein LOC105391939 isoform X3	1	Phospholipase A2	1	/	No	Hydrolases
Hypothetical protein D910_09660	3	Endoribonuclease XendoU	0	S	No	Hydrolases
Uncharacterized protein LOC100164352	1	/	0	/	No	Hydrolases
Hypothetical protein ANCCEY_11605	1	/	0	/	No	Hydrolases
Placental protein 11	1	Endoribonuclease XendoU	1	M	No	Hydrolases
Plancitoxin-1 isoform X1	2	Deoxyribonuclease II	1	S	No	Hydrolases
GA19137 isoform A	1	USP8 dimerisation domain; Rhodanese-like domain; Ubiquitin carboxyl-terminal hydrolase	0	/	No	Hydrolases
N-acetylglucosaminyl-phosphatidylinositol de-N-acetylase	1	GlcNAc-PI de-N-acetylase	1	S	Yes	Hydrolases
Ubiquitin carboxyl-terminal hydrolase 7	1	MATH domain; Ubiquitin carboxyl-terminal hydrolase; ICP0-binding domain of Ubiquitin-specific protease 7; Ubiquitin-specific protease C-terminal	0	/	No	Hydrolases
Uncharacterized family 31 glucosidase KIAA1161 isoform X1	1	Glycosyl hydrolases family 31	0	/	No	Hydrolases
Constitutive coactivator of peroxisome proliferator-activated receptor	1	/	0	/	No	Hydrolases
Endonuclease-reverse transcriptase	1	/	0	/	No	Hydrolases
Neprilysin-11-like isoform X4	1	Peptidase family M13	1	S	No	Peptidases
Xaa-Pro dipeptidase	1	FAST kinase-like protein, subdomain 1	0	/	No	Peptidases
Lon protease-like protein	1	ATP-dependent protease La (LON) substrate-binding domain; ATPase family associated with various cellular activities (AAA); Lon protease (S16) C-terminal proteolytic domain	0	M	No	Peptidases
Serine protease 6	1	Trypsin	1	S	Yes	Proteases
Stubble-2	1	Trypsin	0	/	No	Proteases
Activated CDC42 kinase 1	1	GTPase binding; Protein tyrosine kinase	0	/	No	Transferases
ATP citrate lyase isoform X1	1	ATP-grasp domain; ATP citrate lyase citrate-binding; CoA binding domain; CoA-ligase; Citrate synthase, C-terminal domain	0	/	No	Transferases
Reverse transcriptase	3	Reverse transcriptase (RNA-dependent DNA polymerase)	0	/	No	Transferases
Hypothetical protein D910_10443	1	Reverse transcriptase (RNA-dependent DNA polymerase)	0	/	No	Transferases
Uncharacterized protein LOC105556507	1	/	0	/	No	Transferases
UDP-glucuronosyltransferase 2C1	1	UDP-glucoronosyl and UDP-glucosyl transferase	1	/	No	Transferases
Histone-lysine N-methyltransferase SETMAR	1	/	0	/	No	Transferases
Protein purity of essence isoform X4	1	E3 ubiquitin-protein ligase UBR4	0	/	No	Transferases
RNA-directed DNA polymerase from mobile element jockey-like	1	/	0	/	No	Transferases
Hypothetical protein L798_09082	1	Ring finger domain;CUE domain	0	/	No	Transferases
FAST kinase domain-containing protein 1	1	FAST kinase-like protein, subdomain 2	0	/	No	Transferases
Carbonic anhydrase 2	1	Eukaryotic-type carbonic anhydrase	1	S	Yes	Lyases
Cytoplasmic aconitate hydratase-like	1	Aconitase family (aconitate hydratase); Aconitase C-terminal domain	0	/	No	Lyases
Ornithine decarboxylase	1	Pyridoxal-dependent decarboxylase, pyridoxal binding domain; Pyridoxal-dependent decarboxylase, C-terminal sheet domain	0	/	No	Lyases
Sphingosine-1-phosphate lyase	1	Pyridoxal-dependent decarboxylase conserved domain	0	/	No	Lyases
Protein disulfide-isomerase	1	Thioredoxin	0	S	Yes	Isomerases
ATP-binding domain-containing protein 4	1	Diphthamide synthase; Endoribonuclease L-PSP	0	/	No	Ligases
CTP synthase	1	CTP synthase N-terminus; Glutamine amidotransferase class-I	1	/	No	Ligases
ATP synthase F0 subunit 8	3	/	1	S	Yes	ATP synthases
ATP synthase subunit a	1	ATP synthase A chain	5	S	No	ATP synthases
ATP synthase protein 8	1	ATP synthase protein 8	1	S	Yes	ATP synthases
Vacuolar ATP synthase subunit E	1	ATP synthase (E/31 kDa) subunit	0	/	Yes	ATP synthases
ATP synthase-coupling factor 6	1	Mitochondrial ATP synthase coupling factor 6	0	M	No	ATP synthases
Uncharacterized protein LOC100632426	1	Iron-sulphur cluster biosynthesis	0	/	No	ATP synthases
ATP-binding cassette sub-family G member 4-like protein	3	ABC-2 type transporter; ABC transporter	7	/	No	ABC transporter
ATP-binding cassette sub-family D member 2-like protein	1	ABC transporter transmembrane region 2; ABC transporter	4	/	No	ABC transporter
ATP-binding cassette sub-family B member 7	1	ABC transporter transmembrane region	0	M	No	ABC transporter
ABC protein subfamily ABCH	1	ABC transporter; ABC-2 family transporter protein	5	/	No	ABC transporter
Multidrug resistance-associated protein 1 isoform X1	1	ABC transporter transmembrane region; ABC transporter	16	S	Yes	ABC transporter
Sly1-like protein	1	Sec1 family	0	/	No	Endoplasmic reticulum (ER)–Glogi transporter
Uncharacterized protein LOC100165575	1	/	1	S	No	Ion transporter
Piezo-type mechanosensitive ion channel component 2 isoform X7	1	Piezo non-specific cation channel, R-Ras-binding domain; Piezo	31	S	Yes	Ion transporter
F-box/LRR-repeat protein 7	1	F-box-like; Methyl-CpG binding domain	0	/	No	Ion transporter
Organic cation transporter protein isoform X2	1	Sugar (and other) transporter	10	S	Yes	Ion transporter
Uncharacterized protein LOC105568157	1	/	0	/	No	Ion transporter
Golgin subfamily A member 4	1	GRIP domain	0	/	No	Golgi transporter
Apolipophorin	1	von Willebrand factor type D domain	0	/	No	Lipid transporter
Lipophorin precursor	1	Lipoprotein amino terminal region; Domain of unknown function (DUF1943); Domain of Unknown Function (DUF1081); von Willebrand factor type D domain	0	S	Yes	Lipid transporter
Vitellogenin	1	Lipoprotein amino terminal region; Domain of unknown function (DUF1943); von Willebrand factor type D domain	0	S	Yes	Lipid transporter
Uncharacterized protein LOC100119722	1	Saposin A-type domain; Saposin-like type B, region 1; Saposin-like type B, region 2	2	S	Yes	Lysosome transporter
Vam6/Vps39-like protein	1	CNH domain; Vacuolar sorting protein 39 domain 1; Vacuolar sorting protein 39 domain 2	0	/	No	Lysosome transporter
Vacuolar protein sorting-associated protein 28-like protein	1	VPS28 protein	0	/	No	Lysosome transporter
Run and tbc1 domain-containing protein	1	Rab-GTPase-TBC domain	0	/	No	Lysosome transporter
Hypothetical protein L798_05106	1	Thioredoxin	2	S	Yes	Protein transporter
Charged multivesicular body protein 3	1	Snf7	0	/	No	Vacuolar transporter
Calexcitin-1 isoform X1	1	EF hand; Deoxyribonuclease II	0	S	No;	Calcium ion binding protein
Hypothetical protein TcasGA2_TC004855	1	LETM1-like protein	1	M	No	Calcium ion binding protein
EF-hand domain containing protein	1	EF hand	0	/	No	Calcium ion binding protein
Mucin-like protein	11	/	1	S	Yes	Sheath proteins
Actin	2	Actin	0	/	No	Cytoskeleton protein
Myosin-IIIa	1	IQ calmodulin-binding motif; Myosin head (motor domain)	0	/	No	Cytoskeleton protein
Kinesin light chain	1	Tetratricopeptide repeat	0	/	No	Cytoskeleton protein
Microtubule-actin cross-linking factor 1 isoform X4	1	Spectrin repeat; EF-hand domain pair; Growth-Arrest-Specific Protein 2 Domain	0	/	No	Cytoskeleton proteins
Kinesin-like protein KIF21A isoform X2	1	Kinesin motor domain; WD domain, G-beta repeat	0	/	No	Cytoskeleton proteins
Cordon-bleu protein-like 1	1	/	0	/	No	Cytoskeleton protein
Zinc finger protein 239-like	1	Zinc finger, C2H2 type; C2H2-type zinc finger	0	/	No	Zinc finger protein
Zinc finger matrin-type protein	1	/	0	/	No	Zinc finger protein
Apterous a	1	LIM domain; Homeobox domain	0	/	No	Transcription factors
Ecdysteroid receptor	1	Ligand-binding domain of nuclear hormone receptor; Zinc finger, C4 type (two domains)	0	/	No	Transcription factors
Transcriptional regulator ATRX-like	1	SNF2 family N-terminal domain; Helicase conserved C-terminal domain	0	/	No	Transcription factors
RNA polymerase-associated protein CTR9-like protein	1	Tetratricopeptide repeat	0	/	No	Transcription factors
Conserved hypothetical protein	1	Leo1-like protein	0	/	No	Transcription factors
Transcription initiation factor IIA subunit 1	1	Transcription factor IIA, alpha/beta subunit	0	M	No	Transcription factors
Hypothetical protein L798_07165	1	MH1 domain	0	/	No	Transcription factors
Hypothetical protein L798_07469	1	/	0	M	No	Transcription factors
Elongation factor Tu	1	Elongation factor Tu GTP binding domain; Elongation factor Tu domain 2; Elongation factor Tu C-terminal domain	0	/	No	Translation factors
Elongation factor G2	1	Elongation factor Tu; GTP binding domain	0	M	No	Translation factors
Cryptochrome 2	1	DNA photolyase; FAD binding domain of DNA photolyase	0	/	No	DNA repair and recombination protein
Thyroid receptor-interacting protein 11	1	GRIP domain	0	/	No	DNA repair
Uncharacterized protein LOC103516581	1	/	0	M	No	DNA binding
Cold shock domain-containing protein E1	1	'Cold-shock' DNA-binding domain	0	/	No	DNA binding
HMG box-containing protein 1	1	/	0	/	No	DNA binding
AT-rich interactive domain-containing protein 4B	1	RBB1NT (NUC162) domain; ARID/BRIGHT DNA binding domain	0	/	No	DNA binding
Nucleoprotein TPR	1	/	0	/	No	DNA binding
La-related protein 1B	1	/	0	/	No	mRNA biosynthesis
Mitochondrial fission protein	1	Fis1 C-terminal tetratricopeptide repeat; Fis1 N-terminal tetratricopeptide repeat	1	S	No	Mitochondrial biogenesis
ESF1 homolog	1	NUC153 domain	0	/	No	Nucleotide binding
Bromodomain-containing protein 7	1	Bromodomain; Domain of unknown function (DUF3512)	0	/	No	Protein binding
Bromodomain and WD repeat-containing protein 2	1	/	0	/	No	Protein binding
Chaoptin	1	/	6	M	No	Protein binding
Kazrin-A	1	SAM domain (Sterile alpha motif)	0	/	No	Protein binding
Sorting nexin-17	1	/	0	/	No	Protein binding
Ring finger protein 160	1	Ring finger domain	0	/	No	Ring finger protein
Cisplatin resistance-associated overexpressed protein	1	LUC7 N_terminus	0	M	No	RNA binding
Teneurin-3-like	1	/	0	M	No	RNA binding
R3H domain-containing protein 1 isoform X1	1	R3H domain; SUZ domain	0	/	No	RNA binding
Coiled-coil domain-containing protein 12	1	cwf18 pre-mRNA splicing factor	0	/	No	Spliceosome
Transcription elongation regulator 1	1	WW domain	0	/	No	Spliceosome
Integrator complex subunit 1 isoform X1	1	/	0	/	No	Spliceosome
Anaphase-promoting complex subunit	1	/	0	/	No	Ubiquitin
CD109 antigen-like	1	MG2 domain; Alpha-2-macroglobulin family N-terminal region; Alpha-2-macroglobulin family; Alpha-macro-globulin thiol-ester bond-forming region; A-macroglobulin complement component; A-macroglobulin receptor	0	S	Yes	Antigen
Tubulin-specific chaperone C	1	Tubulin-specific chaperone C N-terminal domain; Tubulin binding cofactor C	0	/	No	Chaperone protein
Interaptin isoform X2	1	/	1	S	Yes	Ribosome-binding protein
Notchless protein homolog 1-like	1	NLE (NUC135) domain; WD domain, G-beta repeat	0	/	No	Ribosome biogenesis
mRNA turnover protein 4 homolog	1	Ribosomal protein L10	0	/	No	Ribosome biogenesis
Hypothetical protein KGM_12879	1	RNA recognition motif. (a.k.a. RRM, RBD, or RNP domain)	0	/	No	Ribosome biogenesis
Putative RNA-binding protein 19	1	RNA recognition motif. (a.k.a. RRM, RBD, or RNP domain)	0	/	No	Ribosome biogenesis
Nucleolar complex protein 3-like protein	1	Nucleolar complex-associated protein; CBF/Mak21 family	0	/	No	Ribosome biogenesis
30S ribosomal protein S2	1	Ribosomal protein S2	0	/	No	Ribosomal protein
39S ribosomal protein L38	1	Phosphatidylethanolamine-binding protein	0	/	No	Ribosomal protein
Uncharacterized protein LOC105558474	1	Ribosomal protein S12/S23	0	/	No	Ribosomal protein
Heat shock cognate 70 protein	1	Hsp70 protein	0	/	No	Heat shock protein
DEP domain-containing protein 5 isoform X4	1	/	0	/	No	Signal transduction protein
Mothers against decapentaplegic homolog 3	1	MH2 domain	0	M	No	Signal transduction protein
Protein numb	1	Phosphotyrosine interaction domain (PTB/PID)	0	/	No	Signal transduction protein
Protein numb-like	1	NUMB domain	0	M	No	Signal transduction protein
Methoprene-tolerant protein	1	PAS domain	0	/	No	Unknown
Salivap-5	1	/	0	/	No	Unknown
Cchamide 1	1	/	1	S	Yes	Unknown
Uncharacterized protein LOC105672384	1	Putative peptidase (DUF1758)	0	/	No	Unknown
Uncharacterized protein LOC105692561	1	/	3	/	No	Unknown
Protein Daple	1	/	0	/	No	Unknown
Rhogtpase	1	BTB/POZ domain	0	/	No	Unknown
Uncharacterized protein LOC105149357 isoform X1	1	/	0	/	No	Unknown
Collagen alpha-1(III) chain	1	/	0	/	No	Unknown
Glutamate receptor ionotropic	1	/	0	/	No	Unknown
Protein retinal degeneration B isoform X3	1	/	0	/	No	Unknown
Hypothetical protein L798_11130	1	/	0	M	No	Unknown
Triple functional domain	1	/	0	/	No	Unknown
Uncharacterized protein LOC100680511	1	/	0	/	No	Unknown
Hypothetical protein TcasGA2_TC010304	1	/	0	/	No	Unknown
Hypothetical protein	1	/	0	S	Yes	Unknown
Uncharacterized protein LOC103508838 isoform X1	1	/	0	/	No	Unknown
Uncharacterized protein KIAA1143 homolog	1	Domain of unknown function (DUF4604)	0	M	No	Unknown
Hypothetical protein L798_03213	1	/	0	/	No	Unknown
Glucocorticoid-induced transcript 1 protein-like	1	Protein Family FAM117	0	M	No	Unknown

^a^ The unique matched peptides are shown in S3 Table.

^b^ Protein domains were determined by Pfam version 31.0 (http://pfam.xfam.org/)).

^c^ THMHH Server v. 2.0 (http://www.cbs.dtu.dk/services/TMHMM/) was used to predict transmembrane helices in proteins.

^d^ Subcelluar localization was predicted by TargetP 1.1 Server (http://www.cbs.dtu.dk/services/TargetP/). M stands for mitochondrial targeting peptide. S stands for secretory pathway signal peptide. “/” stands for other.

^e^Signal peptide was determined by SignalP 4.1 Server (http://www.cbs.dtu.dk/services/SignalP/).

**Fig 1:**
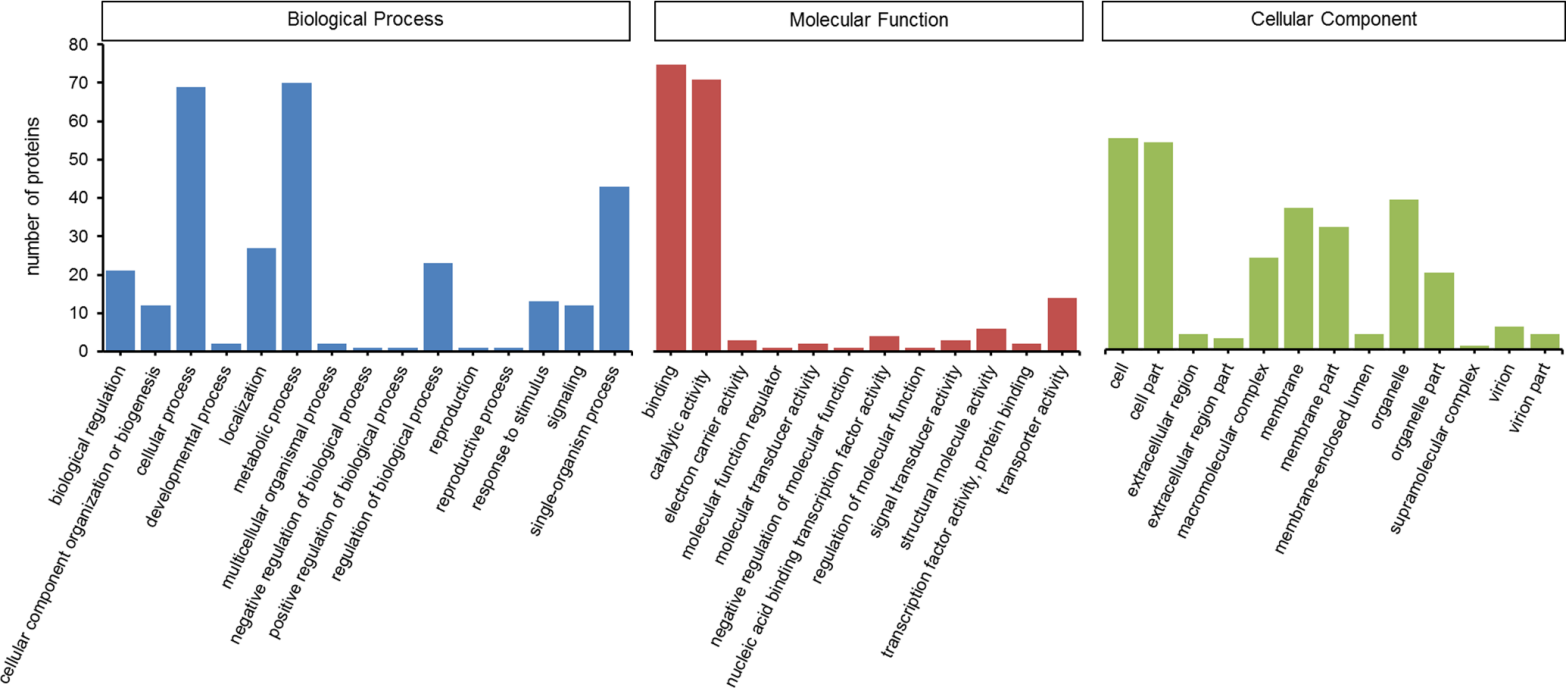
Gene Ontology classification of *S*. *furcifera* water salivary proteins. Saliva components were classified at the second level under three root GO domains: biological process, molecular function and cellular component.

**Fig 2:**
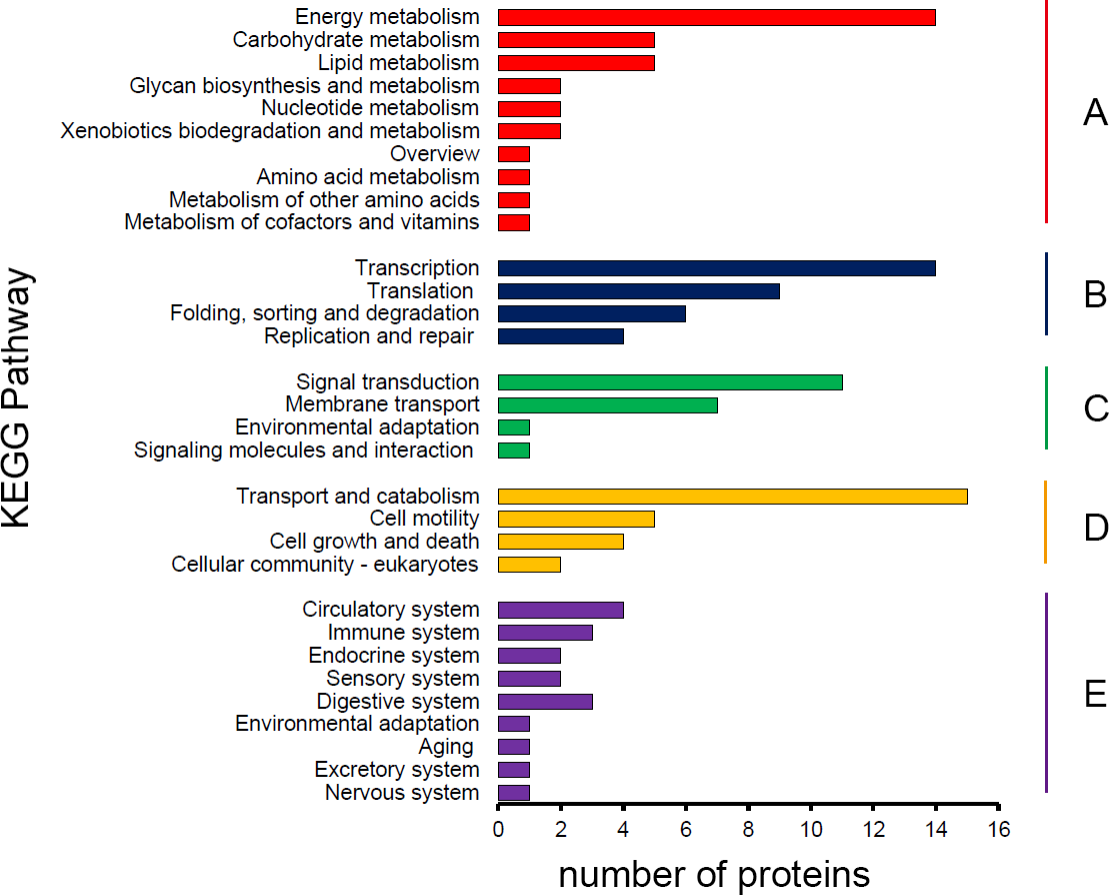
KEGG pathway classification of *S*. *furcifera* salivary proteins. The proteins according to KEGG pathway involved was divided into five branches: A. Metabolism; B. Genetic information processing; C. Environmental information processing; D. Cellular processes; E. Organismal systems.

Gene ontology (GO) and Kyoto Encyclopedia of Genes and Genomes (KEGG) analysis were used to identify the potential functions of *S*. *furcifera* water salivary proteins. For GO annotation, saliva components were classified at the second level under three root GO domains: biological process, molecular function and cellular component (Fig 1). The most two categories were metabolic process (including 70 proteins) and cellular process (69) in biological process, binding (75) and catalytic activity (71) in molecular function, cell (55) and cell part (54) in cellular component. KEGG pathways involved in the salivary proteins were divided into 5 branches: metabolism, genetic information processing, environmental information processing, cellular processes and organismal systems (Fig 2). Most proteins were distributed in metabolism (34) and genetic information processing (33) at the first level, while the majority proteins were related to energy metabolism (14), transcription (14) and transport and catabolism (15) at the second level.

### Predicting, cloning and sequencing of the secretory proteins

Among the 161 proteins, 21 proteins have a putative secretory peptide and no transmembrane domain or the only transmembrane domain was in range of the signal peptide, suggesting secretory proteins. Other proteins without signal peptide indicate unknown secretory mechanism. The putative secretory proteins were searched in the transcriptome of *S*. *furcifera* salivary glands, 11 proteins were found to have the complete ORF, suggesting reliable for sequencing and cloning. Other proteins are not found in the transcriptome or only have a short fragment, so we excluded these proteins. All of the 11 salivary secretory proteins were manually searched by blastx program and then named according to the highest protein similarities with the high amio acid identities range from 54% to 100% in National Center for Biotechnology Information (NCBI) (Table 2). Complete ORFs of 9 proteins were verified by cloning and sequencing, while other two proteins were confirmed with partial ORF. The data were deposited on NCBI (accession number from MF189025 to MF189034) except mucin-like protein, which was the same as the Accession number KX670544.

**Table 2:** Genes of secretory protein identified in *S*. *furcifera* watery saliva.

Gene name	Accession number	Query length (bp)	ORF (aa)	Completeness	Blastx annotation	Score	E-value	Identity
Peptidylglycine α-hydroxylating monooxygenase	MF189025	1044	347	Complete	Peptidylglycine alpha-hydroxylating monooxygenase [*Bactrocera dorsalis*]	459	6e-159	69%
Carboxylesterase	MF189026	1650	549	Complete	Carboxylesterase [*Nilaparvata lugens*]	554	0	54%
Neprilysin-11-like isoform X4	MF189027	1452	483	Complete	Neprilysin-11-like isoform X4 [*Nasonia vitripennis*]	584	0	58%
Serine protease 6	MF189028	819	272	Complete	Serine protease 6 [*Nilaparvata lugens*]	406	2e-141	76%
Carbonic anhydrase 2	MF189029	924	307	Complete	Carbonic anhydrase 2 [*Lygus hesperus*]	303	3e-99	54%
Protein disulfide-isomerase	MF189030	1509	502	Complete	Protein disulfide-isomerase [*Nilaparvata lugens*]	894	0	95%
Vacuolar ATP synthase subunit E	MF189031	681	226	Complete	Vacuolar ATP synthase subunit E [*Nilaparvata lugens*]	378	2e-131	90%
Lipophorin precursor	MF189032	1555	518	Partial	Lipophorin precursor [*Nilaparvata lugens*]	5894	0	86%
Vitellogenin	MF189033	1324	441	Partial	Vitellogenin [*Laodelphax striatella*]	2590	0	89%
Calexcitin-1 isoform X1	MF189034	558	185	Complete	Calexcitin-1 isoform X1 [*Athalia rosae*]	265	6e-88	63%
Mucin-like protein	KX670544	2160	719	Complete	Mucin-like protein [*Sogatella furcifera*]	768	0	100%

### Tissue, development and sex-specific expression analysis

**Fig 3:**
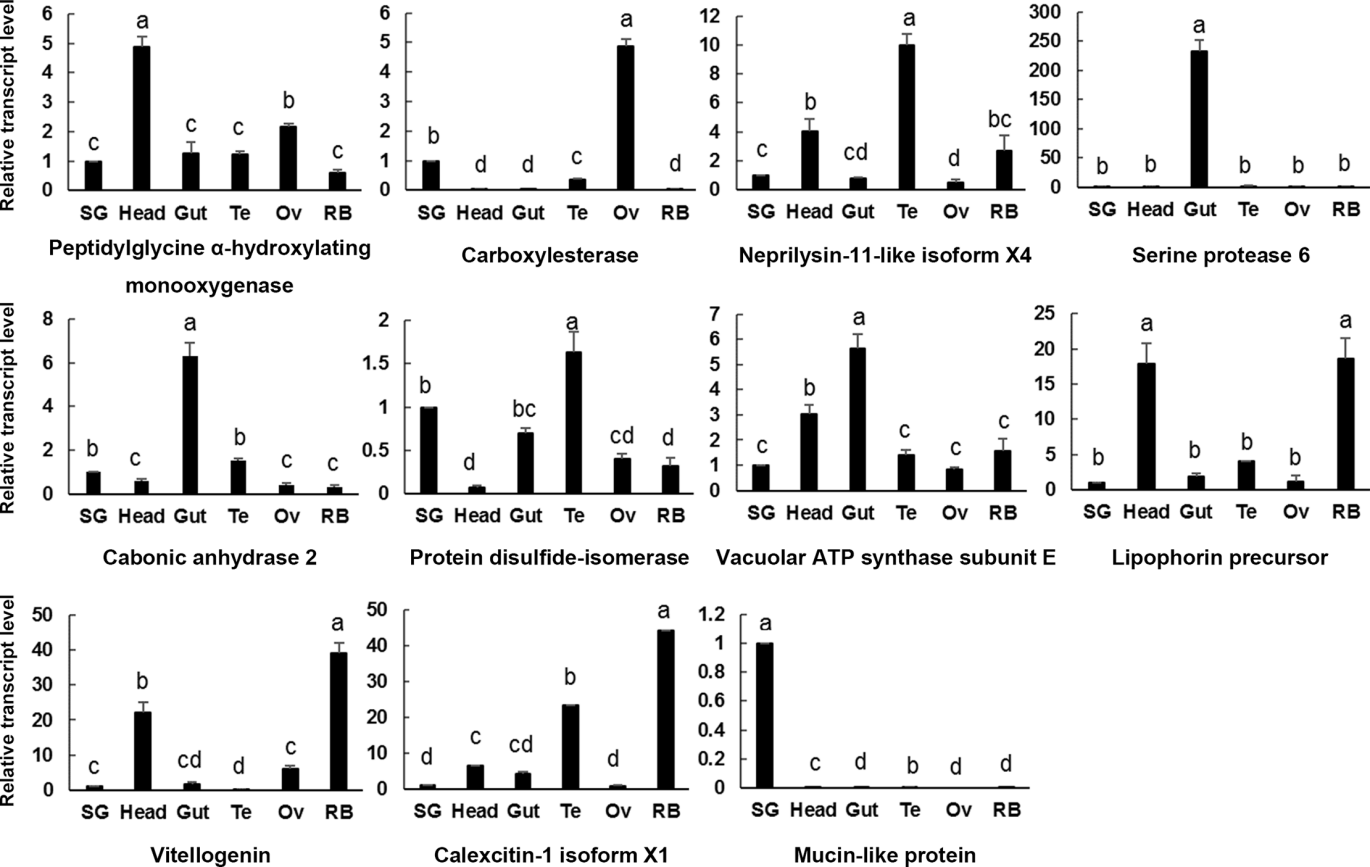
Tissue-specific expression of *S*. *furcifera* genes encoding salivary proteins. SG, salivary gland; Head, head without salivary gland; Gut, gut; Te, testis; Ov, ovary; RB, remaining body.

**Fig 4:**
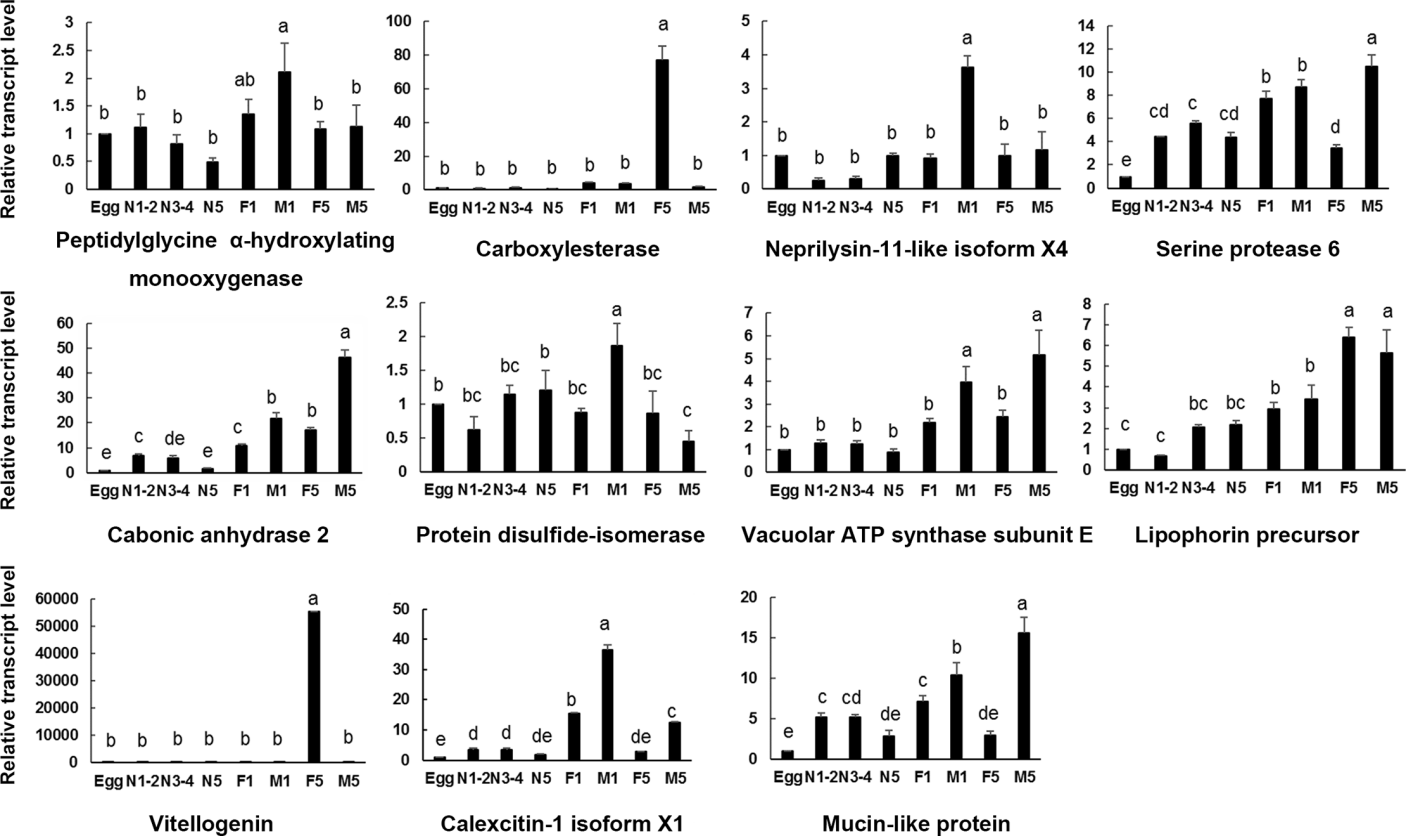
Developmental stage- and sex-specific expression of *S*. *furcifera* genes encoding salivary proteins. Egg, egg period; N1-2, 1^st^-2^nd^ instar nymphs; N3-4, 3^rd^-4^th^ instar nymphs; N5, 5^th^ instar nymphs; F1, newly emerged female adults; M1, newly molted male adults; F5, female adults after molting for 5 days; M5, male adults after molting for 5 days.

The expression of 11 secretory salivary protein genes in different tissues (salivary gland, head, gut, testis, ovary and remaining body) and different developmental stages and sexes (egg, 1^st^-2^nd^ instar nymphs, 3^rd^-4^th^ instar nymphs, 5^th^ instar nymphs, newly emerged female and male adults, and female and male adults after molting for 5 days) were determined by using qPCR. Figs 3 and 4 showed the results.

Oxidoreductases are common enzymes in insect saliva. These enzymes can detoxify phenolic compounds in plant-defense reactions by changing the redox balance [3]. Oxidoreductase peptidylglycine α-hydroxylating monooxygenase was highly expressed in head, followed by ovary. This enzyme was extensively expressed in all developmental stages and no significant difference between female and male.

Hydrolases are related to plant-cell degradation by facilitating stylets penetration and movement in plant cell [3]. Carboxylesterase had the highest transcript level in ovary, followed by salivary gland, and then testis. Other tissues were almost not detected gene expression. This gene was highly expressed in female adult after molting for 5 days, suggesting it may play an important role in reproduction.

Proteases (peptidases) generally function as digestive enzymes and are important in detoxification of plant defense compounds [7]. We investigated two proteases in this study. Serine protease 6 had a unique expression in gut, indicating its digestive function. However, neprilysin-11-like isoform X4 was highly expressed in testis, followed by head and remaining body. The developmental stages and sex specific expression pattern between these two enzymes were also different. The expression level of serine protease 6 was increasing along with the development of insect in general except a decline in female adult. Neprilysin-11-like isoform X4 had a significantly high transcript level in newly emerged male adult.

Lyases degrade substance without hydrolysis and oxidation and lyases in insect saliva were not widely known [3]. Carbonic anhydrase was expressed in all tissues but highly expressed in gut. Adult expression level of this gene was higher than nymph and male expression was higher than female adult. Adult molting for 5 days was expressed higher than newly emerged adult.

We tested an isomerase, protein disulfide-isomerase, which is involved in protein folding and synthesis. This gene was highest expressed in testis and then salivary gland and gut. Protein disulfide-isomerase was expressed in all developmental stages and had the highest expression level in newly emerged male adult. However, the expression decreased significantly in male adult after molting for 5 days, indicating it may function in the male mating with female adult.

ATP synthases are related to energy metabolism. Vacuolar ATP synthase subunit E had an extensive expression in all tissues and developmental phases, but it was expressed higher in gut and head than other tissues. Expression level of this gene in male adult was higher than female.

Lipophorin precursor and vitellogenin are lipid transport proteins that function in modulating immune responses in insects [18, 19]. They share a similar expression pattern in tissues. They were both expressed highly in remaining body and head. As to developmental stages and sexes, they preform differently. Vitellogenin expressed significantly high in female adult molting for 5 days, indicating an important role in female reproduction. Lipophorin precursor expressed in all developmental stages and the expression level was increasing together with the insect development.

Calcium ion binding proteins are recorded in saliva of many aphids. This kind of protein facilitates insects to feed on phloem sap of plants by preventing sieve element occlusion [6]. Calexcitin-1 isoform X1 was mainly expressed in remaining body and testis but hardly expressed in salivary gland. It will be interesting to find how calexcitin enters saliva of *S*. *furcifera*. Calexcitin-1 isoform X1 had a higher expression level in newly emerged male and female adult and male expression was higher than female.

Mucin-like protein was reported to play an integral role in the formation of salivary sheath [20]. The transcript of this protein was detected at significantly high level in salivary gland but was not detectable in head, gut, testis, ovary and remaining body. It was expressed in all stages and sexes while egg period had the lowest expression level and male adult had the highest one.

## Discussion

We investigated the watery saliva component of *S*. *furcifera* in an attempt to understand the interaction between the insects and their host plants. This study has identified 161 proteins which have been classified into 8 categories according to the function. Recently, Huang et al. detected 177 proteins in the secreted saliva of *S*. *furcifera*. After comparison, we found that 10 salivary proteins in our study were the same with Huang’s research, including salivap-5, plancitoxin-1, golgin subfamily A member 4-like, lipophorin precursor, stubble-2, RNA polymerase-associated protein CTR9-like protein, nucleoprotein TPR, AT-rich interactive domain-containing protein 4B, carboxylesterase and vitellogenin. Some similar cytoskeleton proteins like actin, myosin, kinesin-like protein and a transcription factor like elongation factor were also found in our research. However, the majority of identified proteins were different from them, which may be caused by insect geographical population, saliva collection method and LC/MS-MS analysis method. For example, Chaudhary et al. [8] reported saliva of *M*. *euphorbiae* obtained in different diet (0.4% resorcinol, 15% sucrose plus amino acids or water) had different compositions. Our investigated salivary proteins will be useful in increasing the existing knowledge of watery saliva of *S*. *furcifera* and enriching the saliva composition database of *S*. *furcifera*. Some salivary proteins of *N*. *lugens* and *Laodelphax striatellus* that have been found in the study of Huang et al. [13] was also detected in our research, such as carbonic anhydrase, EF-hand domain containing protein, protein disulfide isomerase and mucin-like protein. It is worth mentioning that Huang et al. [20] found the mucin-like protein in *N*. *lugens* was necessary in feeding, especially reared on the resistant rice variety. This protein was confirmed to be involved in the formation of salivary sheath and suppression of resistant plant defense [20].

Since secretory proteins are most likely to be candidate effectors that can secrete into plant tissues and modulate plant defense [21, 22, 23], we selected 11 secretory proteins from the proteomic and transcriptomic analysis for intensive study.

Peptidylglycine α-hydroxylating monooxygenase (PHM), a copper binding oxidoreductase, was highly expressed in the head of *S*. *furcifera* and intensively expressed in all developmental stages. Zabriskie et al. [24] isolated a PHM from heads of honeybees (*Apis mellifera*) and found the enzyme catalyze the amidation of C-terminal peptides, which play important roles in insect reproduction, development and defense. The research about PHM was rarely conducted in invertebrates. The expression pattern about this gene was firstly reported in sap-sucking insects. It may play the same role in formatting of neuropeptides in head as in mammals, and it works in all developmental stages in *S*. *furcifera*.

Carboxylesterases (CarEs) occupy crucial roles in detoxification of xenobiotics, degradation of pheromone, neurogenesis and developmental regulations [25]. We found the CarE was highly expressed in ovary, followed by the salivary gland, suggesting its function in regulation of reproduction and detoxification of plant allelochemicals. The significantly high expression level in female after molting for 5 days also verified its important role in reproduction. CarE was previously detected in the saliva of *N*. *lugens* [4, 12], while the expression pattern was a little different. CarE was expressed in all tissues, with the highest level in salivary gland and the lowest in ovary [12]. This could be because different families of carboxylesterases were detected in saliva of *S*. *furcifera* and *N*. *lugens*.

Peptidases (proteases) are the protein-hydrolysing enzymes which have been reported in many hemipterans [3]. We detected two secreted peptidases in saliva of *S*. *furcifera*. Serine protease 6, acting as digesting and detoxifying enzyme, had a specific expression in gut of *S*. *furcifera* and an extensive expression in all developmental stages except egg. Serine protease was detected in saliva of *Schizaphis graminum* biotype GB-E, GB-G and GB-H other than GB-C (biotype GB-H, GB-G, GB-E, GB-C ranging in virulence from high to low) [6], suggesting its function in detoxification. Serine protease 6 found in saliva of *S*. *furcifera* may play a role in preoral digestion. Neprilysins belong to zinc metalloendopeptidase and play an important role in turning off peptide signaling events at the cell surface [26]. Research on neprilysin and neprilysin-like were conducted on *Locusta migratoria* [27], *Drosophila melanogaster* [28] and *Bactrocera dorsalis* [29], but report about this protein in insect saliva was not found. Neprilysin-11-like isoform X4, detected in *S*. *furcifera* saliva, was highly expressed in testis and newly emerged male adults, suggesting its role in testis development and spermatogenesis. This result was in accord with expression pattern of neprilysin in *B*. *dorsalis* and *D*. *melanogaster*. Neprilysin of *B*. *dorsalis* was specifically expressed in testis [29] and different types of neprilysin in *D*. *melanogaster* were reported to be expressed in malpighian tubules and testis, suggesting roles for the peptidase in excretory function and in spermatogenesis [28]. However, the role of this protein in insect saliva requires further research.

Carbonic anhydrase (CA) and vacuolar ATP synthase were responsible for intracellular pH homeostasis [30]. Slaymaker et al. [31] claimed that tobacco chloroplast carbonic anhydrase binds salicylic acid (SA), indicating function in plant defense response. CA was previously detected in saliva of *S*. *graminum* [6], *Macrosiphum euphorbiae* [8] and *N*. *cincticeps* [10]. It was also found in midgut of larval *Aedes aegypti* [32] and malpighian tubules of *D*. *melanogaster* [33]. CA in *S*. *furcifera* had a highest expression level in gut and relatively high level in testis and salivary gland, while it was specifically expressed in salivary gland in *N*. *lugens* [12]. This may be because CA in different species owns different tissue distribution and function. Vacuolar ATP synthase exists in almost all eukaryotic cells functioning in membrane trafficking, cytosolic alkalinization and extracellular acidification [34]. It was highly expressed in gut and head in *S*. *furcifera*. Hu et al. [35] found that vacuolar ATPase plays an important role in male reproductive physiological processes in *B*. *dorsalis*. CA and vacuolar ATP synthase expression in male adult both were higher than female, indicating more important function in male. These two proteins in saliva of *S*. *furcifera* could involve in the regulation of pH homeostasis.

Protein disulfide isomerase (PDI) is essential for protein folding [36]. PDI was expressed relatively high in testis and salivary gland in *S*. *furcifera*, suggesting its role in regulation of seminal fluid and salivary protein folding. It has also been detected in saliva of *Acyrthosiphon pisum* [9] and *N*. *lugens* [12] previously. Carolan [9] indicated that PDIs were related to an increased yield of salivary proteins. PDI in saliva of *S*. *furcifera* may function in synthesis of salivary proteins.

Lipophorin precursor and vitellogenin (Vg), function as lipid transporter, were found in saliva of *S*. *furcifera*. Lipophorin was reported to act in lipid-based plant defense [6] and previously discovered in water saliva of *S*. *graminum*, *A*. *pisum*, *M*. *euphorbiae* and *N*. *lugens* and *L*. *striatellus* [1, 4, 6, 8, 13]. Vg is known as a nutritional source for embryonic development [37]. It has been reported in gelling saliva of *N*. *lugens* [12]. These two proteins were both expressed highly in remaining body and head. This may be because they were synthesized in fat body, which is abundant in remaining body and head. Lipophorin precursor had a valid expression in all developmental stages while Vg had a significantly high transcript level in female molting for 5 days, indicating its crucial role in reproduction. However, it is still unknown how Vg secretes into the saliva of *S*. *furcifera* and what its role in saliva.

Calcium ion binding proteins exist in saliva of many sap-sucking insects. Innate plant defense mechanism enables occlusion of sieve-tube element when suffering damage by insects [3]. Calexcitin (CE), a calcium ion binding protein, appears as a Ca^2+^-activated signaling molecular [38, 39] and involves in suppressing plant defense. CE had a high transcript level in remaining body and testis, and was highly expressed in newly emerged adults, suggesting a role in regulation of mating and reproduction. CE had the lowest transcript level in salivary gland, leaving us the confusion that how it works in inhibiting of plant defense.

Mucin-like protein was related to the formation of salivary sheath [4], existing in saliva of *N*. *lugens* [4, 12, 13], *L*. *striatellus* [13] and *N*. *cincticeps* [10]. Huang et al. [12, 20] investigated mucin-like protein had a unique expression in salivary gland of *N*. *lugens* and its role in *N*. *lugens* virulence and adaptation to host resistance. Shangguan et al. [40] revealed that mucin-like protein of *N*. *lugens* can induce plant cell death, the expression of defense-related genes and callose deposition. Expression pattern of mucin-like protein was generally the same in *S*. *furcifera*, indicating its function in feeding and interacting with host plant. Male adult had a higher expression than female, suggesting it plays a more important role in male.

In conclusion, we investigated the water saliva component in *S*. *furcifera* and tested the expression pattern of 11 secretory proteins. Many proteins were expressed relatively high in salivary gland of *S*. *fuicifera*, suggesting its role in saliva. While some proteins had a low expression level in salivary gland but were found in saliva, leaving us to find their potential function in saliva. Mucin-like protein was specifically expressed in salivary gland, which is most likely to be an effector functioning in plant defense. Intensive studies are needed to understand the function of this protein.

## Supporting information

S1 Table Primers used for molecular cloning of *S*. *furcifera* salivary protein genes. (XLSX)

S2 Table Primers used in real-time qPCR for gene expression analysis. (XLSX)

S3 Table Unique peptide sequences of *S*. *furcifera* watery saliva proteins identified by LC-MS/MS. (XLSX)
